# Liquid combination of hyaluronan, glucosamine, and chondroitin as a dietary supplement for knee osteoarthritis patients with moderate knee pain: A randomized controlled study

**DOI:** 10.1097/MD.0000000000027405

**Published:** 2021-10-08

**Authors:** Shyu-Jye Wang, Ya-Hui Wang, Liang-Chen Huang

**Affiliations:** a Institute of Medicine, China Medical University, Taichung and Department of Orthopaedics, China Medical University Hospital, Taichung, Taiwan; b Department of Ophthalmology, Taipei Municipal Wanfang Hospital, Taipei, Taiwan; c Department of General Medicine, Chang Gung Memorial Hospital, Taoyuan, Taiwan.

**Keywords:** chondroitin, glucosamine, hyaluronan, knee osteoarthritis, Knee Injury and Osteoarthritis Outcome Score, Western Ontario and McMaster Universities Osteoarthritis Index

## Abstract

**Background:**

Hyaluronan (HA), glucosamine, and chondroitin sulfate are widely consumed as dietary supplements for the treatment of knee osteoarthritis (OA). This study aimed to explore the efficacy and safety of a dietary liquid supplement mixture containing HA, glucosamine, and chondroitin in patients with knee OA who had moderate knee pain (visual analogue scale of 4–6 points).

**Methods:**

This was a short-term, randomized, double-blind, placebo-controlled study. Subjects were allocated to administer either a bottle of 20 mL supplement mixture (50 mg HA plus 750 mg glucosamine plus 250 mg chondroitin, namely A + HA) or placebo once daily for 8 weeks. Outcome measures included the Knee Injury and Osteoarthritis Outcome Score, Western Ontario and McMaster Universities Osteoarthritis Index, 36-item Short Form Survey (SF-36), Chinese version of Pittsburgh Sleep Quality Index, and incidence of adverse event were evaluated at the end of week 8. Efficacy analyses were conducted in the modified intent-to-treat population.

**Results:**

Of the 80 subjects in the modified intent-to-treat population, 39 received A + HA while 41 received placebo. After 8 weeks of treatment, the A + HA group failed to demonstrate a significant symptomatic efficacy and quality of life improvement in terms of Knee Injury and Osteoarthritis Outcome Score, Western Ontario and McMaster Universities Osteoarthritis Index, SF-36, and Chinese version of Pittsburgh Sleep Quality Index as compared to the placebo group. However, the mean changes in most of the SF-36 scale scores were numerically higher in the A + HA group than in the placebo group. No treatment-related adverse event was reported in both groups.

**Conclusions:**

This present study found that the combination of liquid low molecular weight HA, glucosamine, and chondroitin oral supplement did not effectively improve knee OA pain and symptoms after short-term use in knee OA patients with moderate knee pain. However, these results should be interpreted with caution due to the intrinsic limitation of the study design.

## 1 Introduction

Knee osteoarthritis (OA) is the most common joint disease in the adult population. It has a global prevalence of 3.8% in 2010 and its prevalence is rising with age.^[1]^ In addition to age, sex, overweight, obesity, and previous knee injury are also known risk factors for knee OA.^[2,3]^ Currently, a combination of nonpharmacological (exercise, lose weight, physical therapy, or use of walking aids or biomechanical interventions) and pharmacological modalities (oral analgesic, nonsteroidal anti-inflammatory drugs, or intra-articular injections with corticosteroids or hyaluronan [HA]) is recommended for optimal management of knee OA.^[4,5]^

Knee OA is characterized by the progressive breakdown of articular cartilage especially in the weight bearing area. In articular cartilage, aggrecan is a major structural component to provide cartilage its hydrophilicity to resist compression loads. It occurs in the form of proteoglycan aggregate with a HA backbone and high content of chondroitin sulfate chains. A proteoglycan is composed of a core protein with glycosaminoglycan chains. The precursor for the production of glycosaminoglycan is glucosamine.^[6]^ Therefore, it is believed that treatment with HA, glucosamine, and chondroitin sulfate can stimulate the synthesis of proteoglycan, slow the process of articular degeneration, and facilitate joint recovery.^[7]^

As a result, HA, glucosamine, and chondroitin sulfate are widely consumed as dietary supplements in the hope to modify knee OA pathology.^[8]^ Their efficacy and safety have been studied vigorously. A network meta-analysis including 54 studies claimed that glucosamine in combination with chondroitin significantly reduced pain and improved function in knee OA patients than placebo.^[9]^ Positive outcomes of ingested HA in reducing knee pain and function has been demonstrated in a review article.^[10]^ On the basis of the above findings, this study aimed to explore the beneficial effects of a supplement mixture containing glucosamine, chondroitin sulfate, and low molecular weight (5 × 10^4^–5 × 10^5^ Dalton) HA in oral solution form in symptomatic knee OA patients.

## 2 Methods

### 2.1 Ethics

This study was approved by the Institutional Review Board of China Medical University Hospital (DMR101-IRB2-033). The study was conducted in accordance with the International Conference on Harmonization guidelines for Good Clinical Practice (ICH E6) and local legal and regulatory requirements. All subjects provided written informed consent before any study-related procedure was carried out.

### 2.2 Trial design

This was a randomized, double-blind, placebo-controlled, single-center study conducted at China Medical University Hospital, Taiwan. The study was initiated in November 2012 and completed in July 2014. There were total of four visits throughout the 8-week study period. Eligible subjects were randomly assigned to receive either the study product (namely A + HA mixture) or placebo for 8 weeks. Efficacy and safety outcomes were measured at each scheduled visit.

### 2.3 Study eligibility criteria

All subjects were screened for eligibility before enrollment. Only those who met all the inclusion criteria and none of the exclusion criteria were enrolled. The inclusion criteria were male or female ≥40 years of age diagnosed with knee OA grades 1 and 2 based on the definition of Ahlback 1968 and with significant knee OA symptoms within 30 days prior to enrollment. Exclusion criteria were: use of glucosamine within 30 days prior to enrollment; knee OA due to exercise or occupational injury; known allergy to oral HA; had undergone bilateral total knee replacements; wheel chair user; pregnancy; body mass index ≥40 kg/m^2^; diagnosed with cancer; known other causes of arthritis (infectious arthritis, rheumatoid arthritis, connective tissue disease, gout, pseudogout, or psoriatic arthritis); bony or soft tissue malignancy or peripheral neuropathy involving the lower extremities; cardiopulmonary disease which limited walking more than knee pain; had knee instability defined as report of knee buckling or locking within 30 days prior to enrollment; with major neurological deficit that affected gait; with psychiatric illness that limited informed consent giving or Parkinsonism.

### 2.4 Interventions

The study product, A + HA mixture, was a 20 mL oral solution containing a mixture of 50 mg HA (5 × 10^4^–5 × 10^5^ Dalton), 750 mg glucosamine, and 250 mg chondroitin. The placebo was a 20 mL oral solution with similar appearance and odor as the study product but contained no active ingredient. Both the study product and the placebo were manufactured and provided by TOP Pharm. & Medicalware, Taiwan. All eligible subjects were instructed to administer a bottle of study product or placebo once daily in the morning under fasting condition for a period of 8 weeks. No medication was prohibited during the study period. Almost all subjects (96.0%) continued their knee OA treatment and had at least one concomitant medication for knee pain during the study.

### 2.5 Randomization and blinding

A permuted block randomization method with a 1:1 ratio was employed to allocate subjects into one of the two treatment groups. The study conducted in a double-blind manner. Neither the subjects nor the study staffs were aware of the allocation.

### 2.6 Outcomes

The study aimed to assess the effect of A + HA mixture in managing knee OA symptoms. Efficacy was investigated by using the Knee Injury and Osteoarthritis Outcome Score (KOOS), Western Ontario and McMaster Universities Osteoarthritis Index (WOMAC), 36-item Short Form Survey (SF-36), and Chinese version of Pittsburgh Sleep Quality Index (CPSQI). A higher score indicating a better outcome in all efficacy questionnaires except for CPSQI. Safety was investigated through the collection of adverse event (AE) incidence, as well as vital signs monitoring. The primary efficacy point was the changes in KOOS. Secondary efficacy endpoints included the changes in WOMAC, SF-36, and CPSQI. Greater mean change indicating better improvement.

### 2.7 Sample size

The sample size was driven by feasibility, statistical power calculation was not used in establishing the sample size. At least 90 subjects were planned to enroll to achieve at least 40 evaluable subjects per treatment group. Formal hypothesis testing was not performed.

### 2.8 Statistical analysis

Efficacy analyses were based on the modified intent-to-treat population, which defined as randomized subjects who took at least one dose of the treatment product and had at least one post-baseline efficacy evaluation. Safety analyses were based on the safety population, which defined as randomized subjects who received at least 1 dose of the treatment product. A normalized score of 0 to 100 (the higher the score, the better the outcome) was calculated for KOOS, WOMAC, and SF-36. The continuous variable data for demographic, KOOS, WOMAC, SF-36, and CPSQI were analyzed by paired *t* test. For the comparison of the differences between 2 groups, 2-sample *t* test was used. Categorical data for AE were analyzed by Chi-square test or Fisher exact test, as appropriate. All tests were under two-sided 5% significance level.

## 3 Results

**Figure 1:**
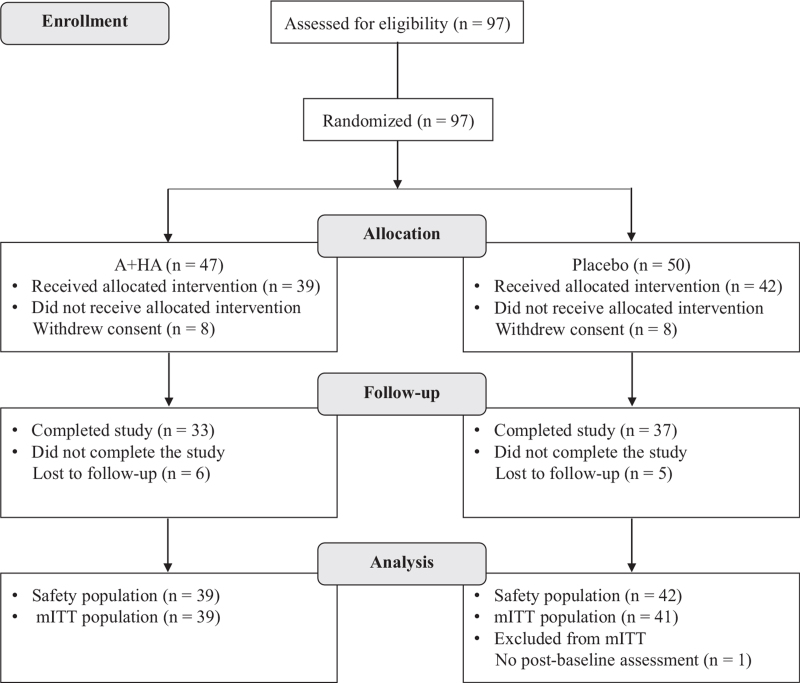
Study flow diagram. HA = hyaluronan, mITT = modified intent-to-treat.

Ninety-seven subjects were screened and randomized. Of these, 81 subjects did receive at least one dose of study product and comprised the Safety population and 80 subjects were included in the modified intent-to-treat population. Eighty-eight percent subjects completed the study in the placebo group, while 85% subjects completed the study in the A + HA group (Fig. 1). The majority of the subjects were female (75%), with a mean age of 59.6 years and mean body mass index of 25.2 kg/m^2^. The mean (standard deviation) visual analogue scale for pain was 5.3 (1.92) in the A + HA group and 5.3 (1.99) in the placebo group. The baseline demographic and clinical characteristics were well balanced between groups and did not differ significantly (Table 1).

**Table 1:** Baseline demographic and clinical characteristics of the mITT population.

Characteristics	A + HA (n = 39)	Placebo (n = 41)	*P* value
Age, yr	58.4 (10.7)	60.8 (9.6)	.28
Sex			.52
Male	11 (28.2%)	9 (22.0%)	
Female	28 (71.8%)	32 (78.0%)	
Weight, kg	66.1 (13.9)	62.4 (11.8)	.20
Height, cm	160.8 (8.1)	157.9 (6.8)	.09
BMI, kg/m^2^	25.5 (4.36)	24.9 (4.07)	.56
KOOS			
Pain	66.8 (17.9)	64.1 (18.9)	.51
Symptoms	49.0 (15.6)	43.6 (17.3)	.14
ADL	69.9 (21.4)	67.3 (20.9)	.59
QOL	51.3 (18.0)	52.6 (16.8)	.74
WOMAC			
Pain	72.7 (18.6)	68.2 (19.7)	.30
Stiffness	61.2 (24.1)	55.2 (25.0)	.28
Function	69.9 (21.4)	67.3 (20.9)	.59
SF-36			
Physical function	45.4 (21.4)	49.5 (22.9)	.41
Role limitation-physical	32.7 (43.0)	42.7 (44.1)	.31
Bodily pain	63.4 (19.7)	62.1 (21.6)	.79
General health	56.4 (18.8)	56.1 (18.4)	.94
Vitality	55.9 (17.2)	58.4 (19.5)	.54
Social function	68.3 (15.9)	66.5 (21.2)	.67
Role limitation-emotional	35.9 (44.8)	50.4 (47.8)	.17
Mental health	63.2 (14.4)	62.7 (20.2)	.91
CPSQI			
Sleep duration	0.7 (0.9)	0.7 (0.7)	.82
Sleep disturbance	1.5 (0.6)	1.5 (0.5)	.83
Sleep latency	1.3 (1.0)	1.4 (1.0)	.48
Daytime dysfunction	1.0 (0.8)	0.7 (0.8)	.11
Sleep efficiency	0.4 (0.9)	0.5 (0.87)	.80
Subjective sleep quality	1.4 (0.7)	1.6 (0.8)	.29
Use of sleep medication	0.9 (1.2)	0.6 (1.1)	.36
Total score	7.1 (3.8)	7.0 (3.2)	.92

Data are presented as mean (SD) or number (%). ADL = activities of daily life, BMI = body mass index, CPSQI = Chinese version of Pittsburgh Sleep Quality Index, KOOS = Knee Injury and Osteoarthritis Outcome Score, mITT = modified intent-to-treat, QoL = quality of life, SD = standard deviation, SF-36 = 36-item Short Form Survey, WOMAC = Western Ontario and McMaster Universities Osteoarthritis Index.

After 8 weeks of treatment, the KOOS dimension scores of pain increased 4.4 (16.4) and 6.4 (16.4), symptoms increased 8.0 (15.8) and 8.6 (19.1), ADL increased 7.3 (18.0) and 7.2 (16.2), and QOL increased 8.5 (17.0) and 6.8 (14.0) from baseline in the A + HA group and the placebo group, respectively. Differences between groups in KOOS did not reach statistical significance (Table 2).

**Table 2:** Changes from 8-week to baseline in KOOS, WOMAC, SF-36, and CPSQI.

	A + HA (n = 39)	Placebo (n = 41)	*P* value
KOOS			
Pain	4.4 (16.4)	6.4 (16.4)	.61
Symptoms	8.0 (15.8)	8.6 (19.1)	.89
ADL	7.3 (18.0)	7.2 (16.2)	>.99
QOL	8.5 (17.0)	6.8 (14.0)	.64
WOMAC			
Pain	3.0 (16.5)	7.2 (17.1)	.31
Stiffness	9.5 (20.7)	12.8 (27.9)	.57
Function	7.3 (18.0)	7.2 (16.2)	>.99
SF-36			
Physical function	11.4 (25.1)	5.4 (24.2)	.32
Role limitation-physical	27.3 (55.7)	10.8 (48.4)	.19
Bodily pain	10.1 (21.8)	6.2 (22.1)	.47
General health	6.5 (16.0)	3.6 (16.4)	.46
Vitality	3.6 (15.3)	5.5 (19.2)	.65
Social function	3.0 (14.7)	1.7 (19.8)	.75
Role limitation-emotional	21.2 (51.9)	3.6 (52.0)	.16
Mental health	−0.4 (15.8)	4.8 (15.7)	.18
CPSQI			
Sleep duration	0.0 (0.4)	0.1 (0.8)	.58
Sleep disturbance	0.0 (0.7)	−0.1 (0.6)	.58
Sleep latency	0.1 (0.9)	−0.2 (0.8)	.08
Daytime dysfunction	−0.2 (0.8)	0.0 (0.9)	.54
Sleep efficiency	0.0 (1.1)	0.1 (1.1)	.67
Subjective sleep quality	−0.1 (0.6)	−0.3 (0.8)	.12
Use of sleep medication	−0.1 (0.9)	−0.1 (0.7)	.94
Total score	−0.2 (3.0)	−0.6 (3.0)	.57

Data are presented as mean (SD). CPSQI = Chinese version of Pittsburgh Sleep Quality Index, HA = hyaluronan, KOOS = Knee Injury and Osteoarthritis Outcome Score, QoL = quality of life, SD = standard deviation, SF-36 = 36-item Short Form Survey, WOMAC = Western Ontario and McMaster Universities Osteoarthritis Index.

For the secondary efficacy endpoints, the WOMAC subscale scores of pain improved 3.0 (16.5) and 7.2 (17.1), stiffness improved 9.5 (20.7) and 12.8 (27.9), and function improved 7.3 (18.0) and 7.2 (16.2) in the A + HA group and the placebo group, respectively, after 8 weeks of treatment. The SF-36 scale scores of physical function improved 11.4 (25.1) and 5.4 (24.2), role limitations due to physical health improved 27.3 (55.7) and 10.8 (48.4), bodily pain improved 10.1 (21.8) and 6.2 (22.1), general health improved 6.5 (16.0) and 3.6 (16.4), vitality improved 3.6 (15.3) and 5.5 (19.2), social function improved 3.0 (14.7) and 1.7 (19.8), role limitations due to emotional problems improved 21.2 (51.9) and 3.6 (52.0), while mental health decreased −0.4 (15.8) and improved 4.8 (15.7) in the A + HA group and the placebo group, respectively, after 8 weeks of treatment. The seven component scores of CPSQI did not change much from baseline after 8 weeks of treatment. The total CPSQI score decreased −0.2 (3.0) and −0.6 (3.0) in the A + HA group and the placebo group, respectively. Differences between groups in WOMAC, SF-36, and CPSQI did not reach statistical significance (Table 2).

As for safety, 22 (27.2%) subjects had at least 1 AE (12 [30.8%] subjects in the A + HA group, 10 [23.8%] subjects in the placebo group), and none of them was treatment-related as judged by the investigator. Upper abdominal pain was the most frequent reported AE in both groups (2 [5.1%] subjects in the A + HA group, 2 [4.8%] subjects in the placebo group). There was no AE that lead to study product discontinuation or study discontinuation.

## 4 Discussion and conclusions

The purpose of our study was to explore whether a short-term intake of a liquid combination of low molecular weight HA, glucosamine, and chondroitin as a dietary supplement could improve the knee OA symptoms and quality of life (QoL) in patients with moderate knee pain (mean visual analogue scale 5.3 ± 1.92). Our findings showed that this combination failed to demonstrate benefit in reducing knee pain or improving knee function. As all patients received oral medications to relieve their knee joint pain in this study, the effect of the study diet supplement or placebo were possibly influenced by the pain medications.

With regards to the symptomatic efficacy as measured by KOOS and WOMAC, the changes at the end of 8-week treatment from baseline were not significant between groups. Subjectively, subjects in the placebo group rated a better improvement in knee pain (mean change: KOOS pain 6.4 vs 4.4; WOMAC pain 7.2 vs 3.0) and symptoms (mean change: KOOS symptoms 8.6 vs 8.0; WOMAC stiffness 12.8 vs 9.5) than the A + HA group, but similar improvement in function as the A + HA group (mean change: KOOS ADL 7.2 vs 7.3; WOMAC function 7.2 vs 7.3). The results were consistent in both KOOS and WOMAC in our study.

As for the QoL, the measurement was conducted using SF-36 and one of the KOOS dimensions. These 2 measurements yielded similar results in the improvement of QoL, that is, subjects in the A + HA group rated a better improvement in the QoL than the placebo group (mean change: KOOS QOL 8.5 vs 6.8). The improvement was greater in the A + HA group than in the placebo group in all SF-36 scales except for vitality and mental health. Interestingly, the results of lack of symptomatic efficacy in pain, symptoms, or function of the A + HA group over the placebo group did not reflect in the SF-36 scales of physical function, role limitation due to physical health, and bodily pain. As all these measurements are subjective, we are not able to draw a possible explanation for these observations.

Nevertheless, several aspects, as well as the limitations, should be mentioned based on these results. First, there was no prohibited medication in our study and all subjects had used oral analgesic or any other medications for knee OA concomitantly due to moderate knee pain during the study period. Therefore, the chance of symptomatic efficacy of the investigatory product overshadowed by the knee OA medications could not be ruled out. Further study that controls the concomitant use of analgesics or assesses the reduction in analgesic use for patients with OA knee pain should be performed for better evaluation of the symptomatic efficacy of the HA, glucosamine, and chondroitin supplement mixture.

Second, the investigational product in our study was a dietary supplement containing 50 mg of HA, 750 mg of glucosamine, and 250 mg of chondroitin. As only one combination was used in this study, it was not known if there is a possibility that the lack of symptomatic efficacy compared to placebo is due to the lower doses of active ingredients used. In a review article published by Reginster et al,^[11]^ numerous studies that demonstrated positive effects of glucosamine in improving pain and function and delaying the structural progression of OA, glucosamine was given at a daily oral dose of 1500 mg. A meta-analysis^[12]^ suggested that chondroitin sulfate at a daily oral dose of 800 mg to 1200 mg has a significant effect in knee OA. Whereas for oral HA, there is still no suggestion in the molecular weight and dose of HA in treating knee OA due to the heterogeneity in current literature. As reported by Guadagna et al,^[13]^ the dosages of HA ranging from 25 mg to 300 mg, while molecular weights ranging from 900 kDa to 2.8 MDa in current literature. Optimal doses of HA, glucosamine, and chondroitin mixture should be focused in future study.

Third, glucosamine, chondroitin, and HA are symptomatic slow-acting drugs for osteoarthritis.^[14,15]^ A longer time study may be needed to achieve a significant beneficial effect in knee OA patients with moderate knee pain. In addition, objective measures such as the presence of radiological progression or actual physical test can be included, given that self-perceived function and actual physical function are different.^[16]^ In addition, the small sample size and single-center design might have introduced selection bias and limited its generalizability. Despite the limitations, this was the first study exploring the efficacy and safety of a dietary liquid supplement combination of HA, glucosamine, and chondroitin in knee OA treatment.

In summary, this present study found that short-term use of oral liquid HA, glucosamine, and chondroitin combination in the current study dose did not effectively improve knee OA pain and symptoms. Future prospective well-designed study with appropriate patient selection and treatment period to find the optimal dose combination is warranted.

## Acknowledgments

We thank all the participants and study staffs of the study.

## Author contributions

SJW designed research, conducted research, and performed statistical analysis; YHW and LCH wrote the paper. SJW had primary responsibility for final content. All authors read and approved the final manuscript.

**Conceptualization:** Shyu-Jye Wang.

**Formal analysis:** Shyu-Jye Wang.

**Funding acquisition:** Shyu-Jye Wang.

**Investigation:** Shyu-Jye Wang.

**Project administration:** Shyu-Jye Wang.

**Writing – original draft:** Ya-Hui Wang, Liang-Chen Huang.

**Writing – review & editing:** Ya-Hui Wang, Liang-Chen Huang.
